# Reduction in labile plasma iron during treatment with deferasirox, a once-daily oral iron chelator, in heavily iron-overloaded patients with β-thalassaemia

**DOI:** 10.1111/j.1600-0609.2008.01204.x

**Published:** 2009-06

**Authors:** Shahina Daar, Anil Pathare, Hanspeter Nick, Ulrike Kriemler-Krahn, Abdel Hmissi, Dany Habr, Ali Taher

**Affiliations:** 1 Sultan Qaboos UniversityMuscat, Oman; 2 Novartis Pharma AGBasel, Switzerland; 3 Novartis PharmaceuticalsEast Hanover, NJ, USA; 4 American University of Beirut-Chronic Care CenterBeirut, Lebanon

**Keywords:** deferasirox, oral, β-thalassaemia, labile plasma iron, pharmacokinetic

## Abstract

This subgroup analysis evaluated the effect of once-daily oral deferasirox on labile plasma iron (LPI) levels in patients from the prospective, 1-yr, multicentre ESCALATOR study. Mean baseline liver iron concentration and median serum ferritin levels were 28.6 ± 10.3 mg Fe/g dry weight and 6334 ng/mL respectively, indicating high iron burden despite prior chelation therapy. Baseline LPI levels (0.98 ± 0.82 μmol/L) decreased significantly to 0.12 ± 0.16 μmol/L, 2 h after first deferasirox dose (*P*=0.0006). Reductions from pre- to post-deferasirox administration were also observed at all other time points. Compared to baseline, there was a significant reduction in preadministration LPI that reached the normal range at week 4 and throughout the remainder of the study (*P*≤0.02). Pharmacokinetic analysis demonstrated an inverse relationship between preadministration LPI levels and trough deferasirox plasma concentrations. Once-daily dosing with deferasirox ≥20 mg/kg/d provided sustained reduction in LPI levels in these heavily iron-overloaded patients, suggesting 24-h protection from LPI. Deferasirox may therefore reduce unregulated tissue iron loading and prevent further end-organ damage.

An excess of iron resulting from regular blood transfusion therapy leads to the appearance of non-transferrin-bound iron (NTBI) in the blood (1). The appearance of NTBI has been hypothesized to increase the risk for developing co-morbidities (2). Labile plasma iron (LPI), a directly chelatable form of NTBI, is readily taken up by cells leading to expansion of the cellular iron pool. As LPI is produced continually in conditions of iron overload, the sustained presence of an iron chelator in the plasma may help avoid accumulation of excess iron, thereby preventing iron-related morbidity and mortality. A limitation of both deferoxamine (DFO) and deferiprone monotherapy is an inability to constantly control levels of LPI (3) because of their short plasma half-lives (4, 5).

Because of to its long half-life of 8–16 h (6, 7), therapeutic levels of the oral chelator deferasirox (Exjade®; Novartis Pharma AG, Basel, Switzerland) are present in the plasma for 24 h following once-daily administration (6). The ESCALATOR trial was initiated to evaluate the efficacy and safety of deferasirox in regularly transfused patients with β-thalassaemia who had previously received chelation therapy with DFO and/or deferiprone. We report here the results of a subgroup analysis examining the effect of deferasirox on LPI levels in a subgroup of patients from a single centre of the ESCALATOR study.

## Patients and methods

### Study design

The ESCALATOR trial was a prospective, open-label, 1-yr, multicentre study conducted in the Middle East. Enrolled patients were males or females aged ≥2 yr with β-thalassaemia. All had transfusional iron overload, as indicated by a liver iron concentration (LIC) ≥2 mg Fe/g dry weight (dw) and serum ferritin levels ≥500 ng/mL, alanine aminotransferase levels <300 U/L and normal renal function at baseline. In addition, all patients had been previously chelated with monotherapy or combination therapy with DFO and/or deferiprone, but chelation was considered unsuccessful because of unacceptable toxicity, contraindication, poor response despite proper compliance or documented non-compliance of taking <50% of prescribed doses in the previous year. Patients who previously received deferiprone discontinued treatment at least 28 d before they received the first dose of deferasirox. Patients were permitted to receive DFO until the day before they initiated deferasirox therapy. Institutional Review Board or Ethics Committee approval was obtained at each participating institution and all patients (or parents/guardians) provided written informed consent. The study was conducted in accordance with Good Clinical Practice guidelines and the Declaration of Helsinki.

All patients included in this analysis received a starting dose of deferasirox 20 mg/kg/d, administered orally once daily. Routine dose adjustments, in steps of 5 or 10 mg/kg/d within a range of 0–30 mg/kg/d, were made according to serum ferritin trends and safety markers. LPI was assessed in a subgroup of 14 patients, all enrolled at a single centre in Oman. Further details of study design and the efficacy and safety results from the full study population have been reported separately (A. Taher, A. El-Beshlawy, M.S. Elalfy, K. Al Zir, S. Daar, G. Damanhouri, D Habr, U. Kriemler-Krahn, A. Hmissi, A. Al Jefri, unpublished data).

### Assessments

Blood samples for LPI assessment were taken preadministration (i.e. the predicted LPI daily peak) and 2 h postadministration (i.e. the predicted daily LPI nadir and approximately equivalent to the time of maximum deferasirox plasma concentration) at baseline (prior to the first deferasirox dose) and following uninterrupted daily administration at weeks 4, 16, 28, 40 and 52. Additional blood samples were taken to evaluate trough and peak plasma concentrations of deferasirox and the deferasirox–iron chelate complex.

LPI levels were analyzed according to the method defined by Esposito (8), using an assay that measures iron-specific redox cycling capacity in the presence of low ascorbate concentrations. Redox reactions were detected by the oxidation of a fluorogenic probe to its fluorescent form. This method allowed distinction of chelator-bound from chelator-free LPI; reported results are from chelator-free LPI.

## Results

### Effect of deferasirox on LPI levels

Overall, 247 of 252 recruited patients into the ESCALATOR trial completed 1 yr of treatment. The LPI subgroup comprised six male and eight female patients with a mean age of 17.5 yr (range 12–27). All 14 patients had previously received DFO/deferiprone combination therapy for a mean period of 30 months (range 9–44). As one patient died because of a non-treatment-related cause (cardiac failure) between weeks 4 and 16, the LPI data presented are from 13 patients.

**Figure 1:**
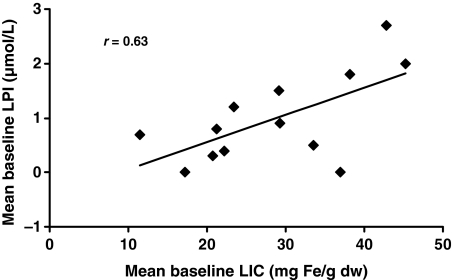
Scatterplot of LPI and LIC at baseline.

**Figure 2:**
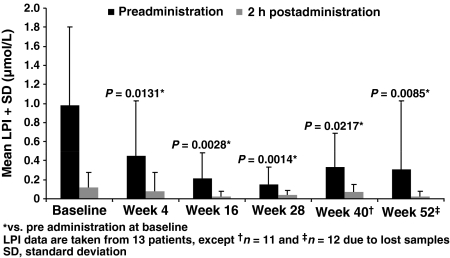
Mean LPI (+SD), pre- and post-administration of deferasirox, throughout the study.

At baseline, mean LIC and median serum ferritin levels were 28.6 ± 10.3 mg Fe/g dw (range 11.5–45.2) and 6334 ng/mL (range 4537–16 620) respectively. Mean baseline LPI levels were 0.98 ± 0.82 μmol/L (range 0–2.7) and were significantly correlated with LIC (*R*=0.63, *P*=0.0198) (Fig. 1). Two hours after deferasirox dosing at baseline, overall mean LPI levels decreased significantly to 0.12 ± 0.16 μmol/L postadministration (*P*=0.0006). A significant reduction in LPI from pre- to post-deferasirox was also observed at weeks 4 (*P*=0.0129), 16 (*P*=0.0269) and 40 (*P*=0.0284); the reduction at weeks 28 (*P*=0.0544) and 52 (*P*=0.1571) was non-significant. There was also a significant reduction in preadministration LPI from baseline to week 4 and week 16, at which point LPI levels were within the normal range (0–0.4 μmol/L) (Fig. 2). Preadministration LPI levels remained significantly lower than baseline and within the normal range at every subsequent time point throughout the remainder of the study. The mean LPI concentrations at week 52 showed a significant correlation with LIC (*R*=0.55, *P*= 0.024). During deferasirox treatment, median serum ferritin decreased by −942 ng/mL (range −4411 to 893) to 5643 ng/mL, while mean (±SD) LIC decreased by −6.8 ± 6.2 mg Fe/g dw (range −14.9 to 3.1) to 21.7 mg Fe/g dw.

### Deferasirox pharmacokinetics

**Figure 3:**
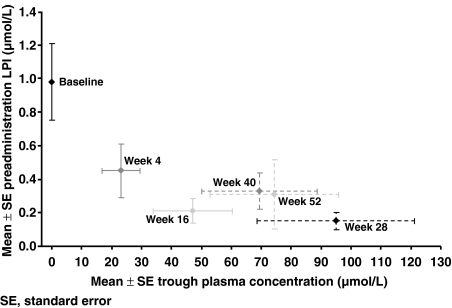
Relationship between preadministration LPI levels (±SE) and trough deferasirox plasma concentration.

Mean steady state plasma concentration (i.e. preadministration value) of deferasirox at weeks 4, 16, 28, 40 and 52 was 23.1, 47.1, 94.9, 69.5 and 74.4 μmol/L respectively (Fig. 3). At each time point, this was approximately half of the concentration value observed at 2 h postadministration (68.8, 116.4, 163.5, 132.0 and 146.4 μmol/L respectively). Figure 3 highlights the relationship between deferasirox plasma concentration and LPI, demonstrating an inverse correlation between preadministration LPI levels and trough plasma concentrations where a plateau appears to be reached at week 16. The deferasirox–iron complex was detectable in the plasma at each time point, with preadministration values ranging from 3.7–9.2 *μ*mol/L.

## Discussion

The results from this 1-yr study highlight the ability of deferasirox ≥20 mg/kg/d to provide sustained reduction in LPI levels. After 4 wk of treatment and throughout the remainder of the 1-yr treatment period, peak LPI levels observed just before deferasirox dosing were significantly decreased compared with baseline; peak LPI levels were within the normal range (<0.4 μmol/L) (3) from week 16 onwards. This is likely because trough levels of deferasirox in this trial are within the therapeutic range, being consistent with those observed in previous trials involving patients of similar ages who received a comparable drug treatment regimen (unpublished data and available by request), thereby preventing LPI levels rebounding between doses. This is in contrast to LPI levels during treatment with other chelators, which have been shown to rebound as chelation coverage decreases between doses (3).

Notably, patients in this subgroup had a high baseline iron burden, despite prior chelation therapy with combined DFO and deferiprone. LIC and serum ferritin levels were markedly higher than thresholds associated with an increased risk of cardiac complications and early death (9, 10). Baseline LPI levels were also high, leading to an increased risk of the production of harmful hydroxyl radicals (8). However, in this subgroup of patients, deferasirox treatment for 1 yr led to reductions in LIC, serum ferritin and LPI.

One patient died because of cardiac failure 1 month after study enrolment; this was not considered to be study drug-related.

In conclusion, once-daily dosing with deferasirox provided sustained reduction in LPI levels in this subgroup of heavily iron-overloaded patients from the ESCALATOR study, suggesting 24-h protection from LPI. Deferasirox may therefore reduce unregulated tissue iron loading and prevent further end-organ damage in these patients.
